# YOLO-LMTB: A Lightweight Detection Model for Multi-Scale Tea Buds in Agriculture

**DOI:** 10.3390/s25206400

**Published:** 2025-10-16

**Authors:** Guofeng Xia, Yanchuan Guo, Qihang Wei, Yiwen Cen, Loujing Feng, Yang Yu

**Affiliations:** School of Mechanical Engineering, Chongqing Three Gorges University, Chongqing 404100, China; 17623097258@163.com (Y.G.); 18996698985@163.com (Q.W.); 18523235914@163.com (Y.C.); 19115674058@163.com (L.F.); 13618694157@163.com (Y.Y.)

**Keywords:** deep learning, tea bud detection, lightweight object detection model, multi-scale feature refinement

## Abstract

Tea bud targets are typically located in complex environments characterized by multi-scale variations, high density, and strong color resemblance to the background, which pose significant challenges for rapid and accurate detection. To address these issues, this study presents YOLO-LMTB, a lightweight multi-scale detection model based on the YOLOv11n architecture. First, a Multi-scale Edge-Refinement Context Aggregator (MERCA) module is proposed to replace the original C3k2 block in the backbone. MERCA captures multi-scale contextual features through hierarchical receptive field collaboration and refines edge details, thereby significantly improving the perception of fine structures in tea buds. Furthermore, a Dynamic Hyperbolic Token Statistics Transformer (DHTST) module is developed to replace the original PSA block. This module dynamically adjusts feature responses and statistical measures through attention weighting using learnable threshold parameters, effectively enhancing discriminative features while suppressing background interference. Additionally, a Bidirectional Feature Pyramid Network (BiFPN) is introduced to replace the original network structure, enabling the adaptive fusion of semantically rich and spatially precise features via bidirectional cross-scale connections while reducing computational complexity. In the self-built tea bud dataset, experimental results demonstrate that compared to the original model, the YO-LO-LMTB model achieves a 2.9% improvement in precision (P), along with increases of 1.6% and 2.0% in mAP50 and mAP50-95, respectively. Simultaneously, the number of parameters decreased by 28.3%, and the model size reduced by 22.6%. To further validate the effectiveness of the improvement scheme, experiments were also conducted using public datasets. The results demonstrate that each enhancement module can boost the model’s detection performance and exhibits strong generalization capabilities. The model not only excels in multi-scale tea bud detection but also offers a valuable reference for reducing computational complexity, thereby providing a technical foundation for the practical application of intelligent tea-picking systems.

## 1. Introduction

With the global proliferation of tea culture, tea has established itself as one of the three most widely consumed beverages worldwide [1]. While mechanical harvesting technologies for bulk tea have matured—typically employing reciprocating cutting mechanisms—they remain unsuitable for premium teas, which require more selective picking to meet higher quality standards [2,3,4]. Moreover, the tea industry is confronted with a shrinking labor force due to an aging population and a lack of interest among younger generations in tea-picking occupations, leading to rising labor costs and significant constraints on industry development [5,6]. These challenges highlight the imperative to develop rapid and accurate tea bud detection technologies.

In recent years, deep learning-based object detection technology has advanced rapidly in the field of image processing. Numerous researchers have employed these methods for detecting tender tea buds. Based on their detection procedures, these approaches can be broadly categorized into two types: two-stage and single-stage detection methods [7]. Two-stage detectors operate under a “localize first, classify later” principle, delivering higher accuracy at the cost of slower inference speeds. Representative algorithms in this category include Fast R-CNN (Fast Region-based Convolutional Neural Network) [8], Faster R-CNN [9], and Mask R-CNN [10]. In contrast, one-stage methods predict both class labels and bounding boxes directly from feature maps in a single forward pass, enabling faster detection while generally achieving slightly lower accuracy compared to two-stage detectors. Well-known one-stage models include the YOLO(You Only Look Once) series [11,12,13,14,15,16,17,18], SSD(Single Shot MultiBox Detector) [19], and RetinaNet [20].

Current methods are primarily based on architectures such as YOLO and R-CNN, where various improvements have been made to enhance detection performance. For example, Yan et al. [21] proposed a Mask R-CNN-based model capable of simultaneously detecting tea buds and locating picking points. The model extends the mask branch to identify tea bud regions by calculating connected areas, determines the principal axis using the minimum enclosing rectangle, and derives picking point coordinates. Experimental results on a self-built dataset show that the model achieves an mAP of 44.9% and an F2-score of 31.3% in tea bud recognition, along with 94.9% accuracy and 91% recall in picking point localization. Li et al. [22] developed a lightweight high-precision object detection model by introducing GhostNet as the backbone of YOLOv4 and replacing standard convolutions with depthwise separable convolutions to reduce computational cost. The model also incorporates CBAM and SIoU loss to improve training efficiency and detection accuracy. Wu et al. [23] proposed a multi-modal tea bud detection model based on YOLOv7, which integrates a parallel depth feature extraction branch and employs a self-attention mechanism for feature enhancement. A cross-modal spatial attention fusion module is introduced to effectively combine depth and RGB features. The model achieves an AP50 of 91.12% in experiments. Li et al. [24] presented GLS-YOLO, an improved YOLOv8-based model, which uses GhostNetV2 as the backbone and depthwise separable convolutions to significantly reduce computational and memory overhead. The C2f-LC module is incorporated to enhance feature recognition through cross-covariance fusion and a lightweight contextual attention mechanism. Additionally, Shape-IoU is adopted as the loss function to improve detection performance for irregularly shaped objects and reduce false positives/negatives. Wang et al. [25] addressed the suboptimal performance of YOLO11 in small object detection scenarios by proposing the PC-YOLO11s detection method. This approach enhances small object feature extraction capabilities, improves detection accuracy, and reduces computational costs by adding a P2 detection layer, removing the P5 layer, and introducing a coordinate space attention mechanism. To thoroughly validate the effectiveness of these improvements, experiments were conducted on both the VisDrone2019 and Tea Bud datasets. Results demonstrate that PC-YOLO11s achieves overall superior performance compared to other existing models in the YOLO series.

However, several key challenges remain in this field. Most existing tea datasets are primarily captured from a top-down perspective focused on the tea canopy, resulting in limited scale variation that struggles to accommodate the significant multi-scale differences characteristic of vertically growing tea varieties. Moreover, tea buds often occur in dense clusters within complex environments where low foreground-background contrast further degrades detection performance. Finally, deploying detection models on edge devices necessitates lightweight architectures, creating an ongoing challenge to balance high accuracy with computational efficiency.

To address the aforementioned challenges, this paper proposes YOLO-LMTB, a tea bud detection model based on the YOLOv11n architecture. The main contributions are as follows:

(1) A Multiscale Edge-Refinement Context Aggregator (MERCA) module is proposed to capture and integrate multi-scale contextual information, constructing rich feature representations. It incorporates an edge-aware refinement mechanism that enhances critical structural details—such as bud contours, tips, and leaf junctions—while suppressing background noise for clearer edge delineation.
(2) A Dynamic Hyperbolic Token Statistics Transformer (DHTST) module is designed, which uses a learnable Tanh threshold to dynamically modulate feature responses. This enhances activations in target regions while suppressing areas with low signal-to-noise ratio. Token-wise statistical attention weighting further strengthens discriminative features and reduces background interference.
(3) A Bidirectional Feature Pyramid Network (BiFPN) is introduced to replace the original fusion structure. It establishes bidirectional cross-scale connections, enabling deeper integration of multi-level features. This enhances both semantic and spatial representation while reducing computational redundancy, leading to a lighter and more efficient model.
(4) A complex tea bud detection dataset is constructed. Reflecting real harvesting conditions, images were captured from multiple angles to cover scale and perspective variations in vertically grown tea buds. The dataset is carefully annotated following the “one leaf, one bud” standard for premium tea, providing high-quality labels to support model training and evaluation and facilitate further research.

The remainder of this paper is structured as follows. Section 2 details the dataset construction and the architecture of YOLO-LMTB, particularly analyzing the proposed model improvements. Section 3 describes the experimental setup, presents results from comparative and ablation studies, and further validates the effectiveness of the proposed method through visualization of the results. Finally, Section 4 concludes the paper and discusses limitations along with directions for future research.

## 2. Materials and Methods

### 2.1. Tea Bud Self-Built Dataset and Public Dataset

The Self-built dataset used in this study was collected from the Xiaoshui Tea Plantation in Yujia Town, Wanzhou District, Chongqing, China, focusing on the “Golden Bud” tea variety—known as the “panda of teas” for its high economic value, with market prices ranging from 3000 to 5000 CNY per 500 grams. Due to the strong seasonality of tea harvesting, image acquisition took place between March 25 and 31, 2025, from 7:00 AM to 5:30 PM daily, to incorporate variations in lighting and weather conditions. A Canon R100 DSLR camera with an APS-C CMOS sensor was used to capture images at a resolution of 6000 × 4000 pixels, with a bit depth of 24 bits. To reflect the vertical growth pattern of Golden Bud tea and simulate real picking scenarios, images were taken from distances of 30–100 cm and camera angles ranging from 0° to 50° relative to the overhead position. After rigorous screening, 3062 images were selected for the final dataset.

**Figure 1:**
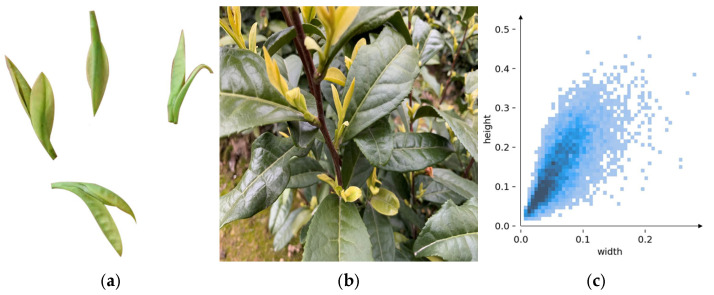
Example of Tea Bud Dataset and Scale Distribution. (**a**) Tea farmers picking tea leaf samples; (**b**) Sample Photography; (**c**) Scale distribution of tea labels in the dataset.

Through interviews with local tea farmers and consultations with the plantation owner, it was determined that only tea shoots meeting the “one leaf, one bud” standard are harvested in practice to ensure both quality and yield. This standard was strictly followed during the image annotation process. The annotation software LabelImg (Version: 1.8.6) was used to generate TXT files containing tea category labels and bounding box coordinates. The dataset was divided into training set (2143 images), test set (613 images), and validation set (306 images) in a 7:2:1 ratio. In subsequent experiments, Mosaic online data augmentation was applied to further enhance the diversity of the training set (2143 images). Figure 1 illustrates tea farmers harvesting tea and the distribution of tea target scales within the dataset.

To further validate the effectiveness of the model improvement scheme, we conducted additional experiments on the publicly available tea bud dataset [26] in addition to various experiments based on our self-built dataset. This public dataset comprises 5000 images, divided into a training set (3500 images), a test set (500 images), and a validation set (1000 images) at a ratio of 7:1:2. Similarly, we continued to employ Mosaic for online data augmentation of the training set, maintaining the same augmented training set size.

### 2.2. Improved Method

**Figure 2:**
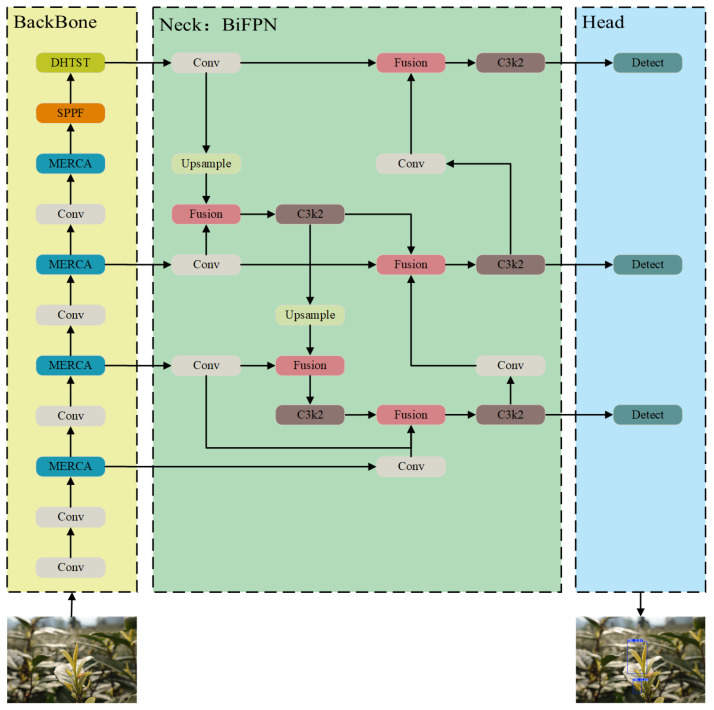
The network structure of YOLO-LMTB. The image in the lower left corner displays the model input information. The blue border in the image in the lower right corner indicates the model detection output results.

Similarly to mainstream object detection frameworks, the proposed YOLO-LMTB model consists of a backbone network, a feature fusion neck, and a detection head [27], as illustrated in Figure 2. 

To address the challenges posed by the uneven distribution and multi-scale variations in tea buds in real scenes, we enhance the original C3k2 feature extraction module. The improved module captures multi-scale contextual semantic information in parallel and incorporates edge refinement to enhance the representation of fine structural details across diverse targets. Furthermore, to improve the fusion of tea-related features, we propose a DHTST module to replace the original C2PSA module in the neck network. Finally, considering the computational constraints of edge deployment, we introduce an efficient BiFPN to entirely replace the original PAFPN structure. This replacement not only improves feature integration but also significantly reduces computational complexity.

#### 2.2.1. Multi-Scale Edge-Refinement Context Aggregator (MERCA)

In natural tea plantation environments, the biological characteristics of tea buds and their interactions with complex field conditions are the main factors limiting detection performance. Tea buds display typical multi-scale morphological continuity during growth: within the same period, tightly wrapped tips measure around 3–5 mm, while partially unfolded young leaves can extend up to 2 cm. For vertically growing tea varieties, variations in imaging angle inevitably introduce scale differences during acquisition. This wide range of scales makes it difficult for conventional convolutional networks with fixed receptive fields to capture comprehensive features, particularly resulting in low recognition rates for tiny bud tips. Moreover, the waxy cuticle on young buds reflects natural light, and overlapping branches and leaves combined with high-density growth often cause shadow occlusion. These factors collectively lead to blurred bud-leaf boundaries and further complicate accurate detection.

**Figure 3:**
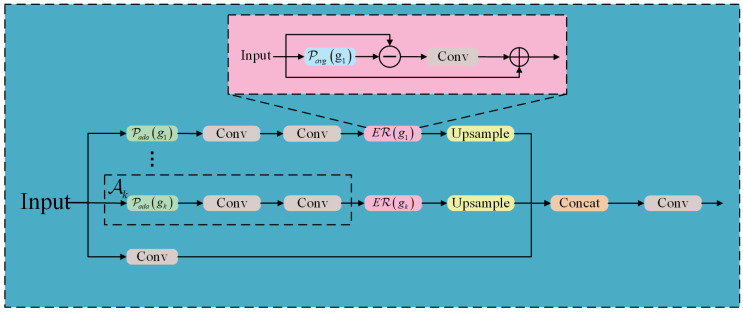
The structure of MERCA and ER.

To address the aforementioned challenges, this paper proposes a Multi-scale Edge-Refinement Context Aggregator (MERCA) module to replace the original C3k2 feature extraction block. The MERCA module enhances the model’s capacity for simultaneous multi-scale feature capture and edge detail refinement in tea bud detection. It adopts a collaborative architecture that decouples multi-scale features and refines edge structures to achieve multi-scale edge perception. The structure of the module is illustrated in Figure 3, and its computational process is described as follows:

(1) Multi-scale Feature Decoupling: Upon entering the module, the feature map is processed by multi-scale receptive field units tailored to tea bud dimensions to form a feature pyramid (e.g., $G=\left\{g_{k}|g_{k}=3k,k\in \left[1,4\right]\cap \mathbb{Z}\right\}$). The input features are mapped into different scale spaces via adaptive average pooling. Each scale-specific branch employs a cascaded convolutional structure acting as a scale adapter, enabling scale-aware feature decoupling through a parameterized pyramid architecture. This design effectively tackles the challenge of cross-scale feature extraction in tea bud detection. The multi-scale adapter $A_{k}$ is formulated as follows:

(1) $$A_{k}=Conv_{3\times 3}^{g}\left(Conv_{1\times 1}\left(P_{ada}\left(Xg_{k}\right)\right)\right)$$

where $Xg_{k}$ denotes the feature input of the k-th scale space, $P_{ada}$ represents adaptive average pooling, and $Conv_{3\times 3}^{g}$, $Conv_{1\times 1}$, respectively, denote the 3×3 grouped convolution and 1×1 dimensionality-reduction convolution.

(2) Edge Feature Refinement: The input features are first smoothed via average pooling to extract low-frequency components. High-frequency edge information is then isolated through differential operations. These high-frequency components are subsequently enhanced using convolutional layers and activation functions to accentuate edge details. Finally, feature reconstruction is performed via residual connections. Through this process, the refined tea bud features preserve the original semantic information while capturing multi-scale edge details, thereby improving discriminability for subsequent processing. The edge feature refiner $ER\left(X\right)$ is formulated as follows:

(2) $$ER\left(X\right)=X+Conv\left(X-P_{avg}(X)\right)$$

where X represents the input feature, and $P_{avg}$ denotes average pooling.

(3) Context Aggregation Mechanism: Following the CSP design philosophy, multi-scale decoupling is applied to tea bud features while retaining local context through parallel convolutions that process the original-resolution features to preserve fine-grained details. The decoupled multi-scale features are resized to the original dimensions via bilinear interpolation and concatenated along the channel dimension to achieve contextual aggregation. Finally, convolutional layers enable cross-scale feature interaction, forming a unified multi-scale tea bud edge feature perception system. This mechanism supplies more discriminative features for subsequent detection tasks and is critical to ensuring prediction accuracy. The output of the context aggregation mechanism is formulated as follows:

(3) $$Y=Conv_{agg}\left(X_{fusion}\right)\in {\mathbb{R}}^{C\times W\times H}$$

where Y denotes the final output feature, $X_{fusion}$ represents the concatenation of multi-scale features and local features, and $Conv_{agg}$ signifies the context aggregation convolution.

In summary, the overall formula for the MERCA module can be expressed as:

(4) $$Y=Conv_{agg}\left(Concat\left(Conv_{local}\left(X\right),\sum_{k=1}^{N}ER_{k}\left(A_{k}\left(Xg_{k}\right)\right)\right)\right)$$

where $Conv_{local}$ denotes parallel convolutions processing features at the original resolution, and *Concat* represents the dimension concatenation operation between features from the first to the *N*-th scale and the original features.

Finally, the MERCA module is designed to tackle key challenges in complex tea growth environments, such as multi-scale feature extraction and blurred high-frequency edge information. It establishes a multi-branch feature extraction pathway, enhances boundary representation through a dedicated edge refinement mechanism, and integrates cross-scale contextual features. The module strengthens the model’s ability to characterize multi-scale tea bud structures and significantly improves robustness in challenging scenarios.

#### 2.2.2. Dynamic Hyperbolic Token Statistics Transformer (DHTST)

In the task of detecting tender tea buds in complex agricultural environments, enhancing and optimizing feature representations is essential for improving detection accuracy. Current methods primarily rely on static activation functions to model the nonlinear relationship between input signals and complex outputs. However, such functions lack adaptability to the intricate conditions of tea fields, limiting the model’s ability to distinguish low-contrast features between tender buds and the background. Furthermore, conventional attention mechanisms depend heavily on feature similarity computations. In tea plantation scenarios, the high similarity between foreground and background often leads to false activations.

**Figure 4:**
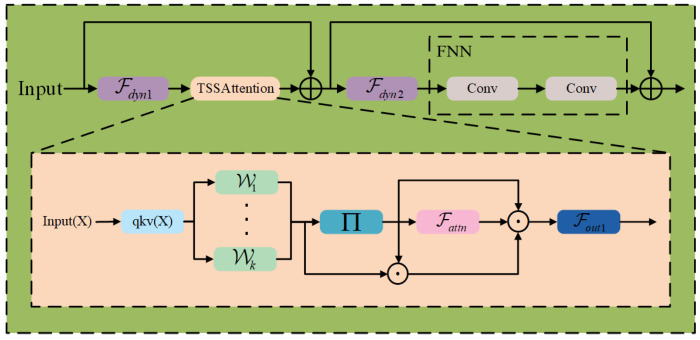
The Structure of DHTST Module.

To tackle the challenges mentioned above, we propose the Dynamic Hyperbolic Token Statistics Transformer (DHTST) module, which integrates the Dynamic Tanh (DyT) [28] and a Token Statistical Self-Attention (TSSA) mechanism [29]. The structure is shown in Figure 4. Deployed at key locations between the feature extraction backbone and the neck network, this module dynamically modulates feature responses via a learnable hyperbolic transformation layer. The feature statistical recalibration mechanism computes attention weights based on token-wise distribution characteristics, while a nonlinear feedforward network with residual connections enables collaborative feature space mapping. This design overcomes the limitations of conventional attention mechanisms by preserving high-frequency edge details and suppressing background noise.

The DHTST module functions as a progressive feature enhancement mechanism, operating through the following processing stages: 

(1) Feature Dynamization: As the core preprocessing stage of the DHTST module, the Feature Dynamization layer addresses sensitivity issues such as lighting variations and inconsistent feature distributions in tea bud detection. The input features are first split into two branches. One branch undergoes parameterized hyperbolic transformation within this layer, enabling adaptive nonlinear mapping of the input features and dynamically adjusting the feature response range. By compressing the input features into the interval [−1, 1] using the DyT operator, outlier feature values are effectively suppressed. The Feature Dynamization process $F_{dyn}$ is formulated as follows:

(5) $$F_{dyn}=\beta ⊙\tanh\left(\alpha ⊙X\right)+\gamma$$

Among these, α,β,γ represent the learnable scaling factor, channel weight, and bias compensation, respectively.

(2) Feature Statistical Recalibration: This layer is designed to overcome the limitations of conventional attention mechanisms in tea bud detection, such as insufficient feature statistics modeling and poor adaptability. Traditional attention mechanisms compute weights based on feature similarity, which in complex environments makes them susceptible to distraction from semantically similar backgrounds and less effective at distinguishing targets. Furthermore, fixed attention patterns fail to adapt to the distributional characteristics of targets across different scales. In this layer, the dynamically activated features are reorganized spatially, and their second-order statistics are incorporated to achieve adaptive attention weight allocation. This process projects the features into a low-dimensional subspace, suppresses irrelevant feature directions, and reduces background interference. The mathematical formulation is as follows:

(6) $$W_{k}=rearrange(q,k,v(F_{dyn1}))\in {\mathbb{R}}^{b\times h\times n\times d}$$

(7) $$\Pi =\mathrm{soft}\max\left(\left(\sum_{i=1}^{k}{\left(\|W_{i}{\|}_{2}\right)}^{2}\right)\cdot \tau \right)\in {\mathbb{R}}^{b\times h\times n}$$

(8) $$F_{attn}=dropout\left({\left((\frac{\Pi }{\sum_{n}\Pi +\varepsilon })\cdot W^{2}+1\right)}^{-1}\right)$$

(9) $$F_{out1}=\left(droupout\left(linear\left(rearrange\left(-\left(\left(W⊙\Pi \right)⊙F_{attn}\right)\right)\right)\right)\right)\in {\mathbb{R}}^{b\times n\times \dim}$$

where $W_{k}$ denotes feature rearrangement into a multi-head format, Π represents attention weights, τ denotes learnable parameters, $F_{attn}$ denotes attention outputs, and $F_{out1}$ denotes the output after feature fusion.

(3) Multi-scale Feature Fusion: The output features from the auxiliary branch, after dynamic activation and statistical recalibration, are concatenated with the original features from the main branch along the channel dimension. The combined features are then split into two pathways. One pathway undergoes secondary dynamic activation for nonlinear enhancement and is subsequently fused with the main branch features via a Feedforward Network (FFN) layer. This procedure enhances representational capacity while preserving original feature integrity, resulting in the desired effect of “feature preservation and depth enhancement.” The first feature fusion step $F_{fusion1}$ can be expressed as:

(10) $$F_{fusion1}=X⊕\left(F_{out1}\left(F_{dyn1}\left(X\right)\right)\right)$$

In summary, the overall formula for the DHTST module can be expressed as:

(11) $$Y=F_{fusion1}⊕\left(Conv_{ffn}\left(F_{dyn2}\left(F_{fusion1}\left(X\right)\right)\right)\right)$$

where $Conv_{ffn}$ denotes the convolution of the two FFN layers.

Overall, the DHTST module enhances its ability to represent tea leaf features through the synergistic optimization of dynamic activation functions and feature statistical recalibration mechanisms. This enables the network to precisely focus on the characteristics and spatial distribution of tender tea buds within complex tea garden environments, thereby improving the model’s detection accuracy.

#### 2.2.3. Bidirectional Feature Pyramid Network (BiFPN)

The neck network in YOLOv11 improves detection accuracy by integrating multi-scale feature maps from the backbone. However, its information flow is predominantly unidirectional or relies on simple bidirectional aggregation, often resulting in the dilution or loss of shallow features during top-down propagation. Moreover, effective feature fusion typically necessitates a deeper neck structure, increasing computational burden and hindering deployment efficiency on edge devices.

**Figure 5:**
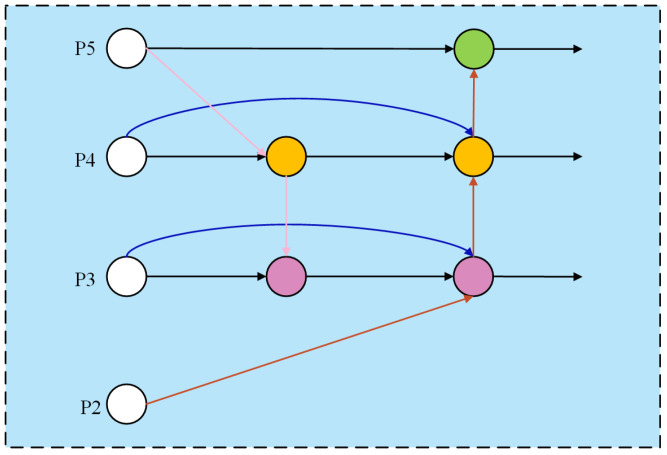
The structure of BiFPN.

To address these issues, we introduce the Bidirectional Feature Pyramid Network (BiFPN) [30] as the neck structure, and its structure is shown in Figure 5. This architecture is designed to reduce computational complexity and enhance multi-scale feature fusion through efficient bidirectional cross-scale connections and adaptive weighted fusion. It effectively preserves and integrates high-resolution details from shallow layers, emphasizing discriminative features of tea targets while eliminating redundant components. The workflow is as follows:

The original feature maps (P3, P4, P5) from the backbone network first undergo convolutional operations for channel compression, unifying dimensionalities across scales. This preprocessing step reduces computational complexity while retaining spatial information. The P5 features are then upsampled and fused with P4 via weighted feature fusion. The fused output is enhanced through three convolutional layers to generate new P4 features, which are further upsampled and merged with P3 to form a semantically enriched shallow feature representation—effectively propagating high-level semantics to shallower layers. Next, P2 features from the backbone are introduced and downsampled to the P3 scale to supply fine edge details of tea buds. The original P3 features, top-down enhanced features, and high-resolution P2 features are adaptively fused through weighting. Finally, the fused features are downsampled and propagated sequentially to P4 and P5, completing the bidirectional information flow. The detection head receives the refined P3, P4, and P5 feature maps, achieving high-precision tea bud detection along with model lightweighting.

## 3. Experimental Results and Analysis

### 3.1. Evaluation Criteria

To comprehensively evaluate the performance of the proposed model in tea bud detection, this study focuses on two key aspects: detection accuracy and model efficiency. Detection accuracy is assessed using precision (P) and mean average precision (mAP), while model efficiency is evaluated based on parameter count, FLOPs (floating point operations), and model size. The definitions of these metrics are as follows:

(12) $$P=\frac{TP}{TP+FP}$$

(13) $$R=\frac{TP}{TP+FN}$$

(14) $$mAP=\frac{\sum_{n=1}^{N}\int_{0}^{1}P_{n}\left(R_{n}\right)dR_{n}}{N}$$

True positives (TP) denote correctly detected target instances, false positives (FP) refer to erroneous detections, and false negatives (FN) indicate missed ground-truth instances. Precision (P) reflects the proportion of correct predictions among all detected samples, measuring the model’s prediction accuracy. The mean average precision (mAP) incorporates both precision and recall (R), offering a comprehensive assessment of the model’s overall detection capability. A higher mAP signifies better detection performance.

### 3.2. Experimental Environment

To ensure the accuracy and scientific validity of the experimental results, all subsequent ablation and comparison studies were conducted under the following hardware and parameter configurations. The experimental environment used Windows 11, Python 3.10, PyTorch 2.3.0, and CUDA 12.1. Training was performed on a system equipped with a 12th Gen Intel^®^ Core™ i5-12600KF CPU (3.70 GHz), an NVIDIA GeForce RTX 4070 Super 12GB GPU, and 32 GB of RAM (Santa Clara, CA, USA).

The training hyperparameters were set as follows: input images were resized to 640 × 640 pixels; the model was trained for 300 epochs with a batch size of 32 and 4 worker threads; the initial learning rate was 0.01, weight decay was 1 × 10^−5^, momentum was 0.973, and stochastic gradient descent (SGD) was used as the optimizer.

### 3.3. Comparative Experiment

#### 3.3.1. Analysis of MERCA’s Effectiveness

This paper addresses the issue of diverse target scales in tea leaves by proposing the MERCA module, which replaces the C3k2 module in the original YOLOv11 model to enhance performance. However, the original model contains multiple instances of the C3k2 module. To analyze how the placement of the MERCA module affects model performance, this study compares three replacement scenarios: replacing the C3k2 module in the backbone network, replacing the C3k2 module in the neck structure, and replacing all C3k2 modules in the network. The improved models are named YO-LO11n-MERCA-Backbone, YOLO11n-MERCA-Neck, and YOLO11-MERCA-All, respectively. Comparative experiments were conducted using a self-built tea bud dataset, with results shown in Table 1.

Table 1 shows that replacing the C3k2 module in the backbone network with the MERCA module reduces model precision but improves mAP50 and mAP50-95 metrics without increasing model parameters or volume. The other two replacement schemes failed to enhance model performance, particularly when replacing the C3k2 module in the neck network, where detection capability significantly weakened. This is because the MERCA module exhibits high sensitivity to target edge information. When deployed in the backbone network, this module assists the model in achieving superior feature extraction. However, when placed after the neck network where feature abstraction has already been completed, its edge sensitivity becomes a negative optimization factor, leading to decreased detection accuracy. Experimental results ultimately confirm that replacing the C3k2 module in the backbone network with the MERCA module is the optimal optimization strategy. All subsequent improvement schemes adopt this approach.

#### 3.3.2. Comparative Experiment of Mainstream Models

To evaluate the effectiveness of the proposed improvements, we compared our model against several representative object detection algorithms, including YOLOv5n, YOLOv8n, YOLOv10n, YOLOv11n, YOLOv12n, Hyper-YOLO [31], and YOLOX-tiny [32]. Additionally, two parameter configurations of YOLOv11 were included for a more comprehensive comparison. All models were tested on the Self-built dataset, and the experimental results are presented in Table 2.

**Figure 6:**
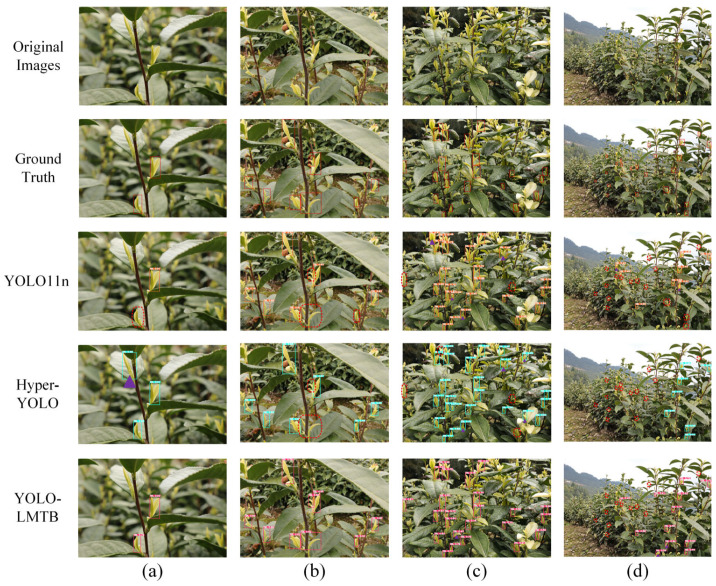
Detection results visualization of YOLO11n, Hyper-YOLO, and YOLO-LMTB. (**a**) Scenes with few targets, no occlusions, and simple backgrounds; (**b**) Scenes with moderate targets, occlusions, and simple backgrounds; (**c**) Scenes with numerous targets, occlusions, and complex backgrounds; (**d**) Scenes with extremely small targets. Red dashed boxes indicate missed targets; purple triangles indicate false positives.

**Figure 7:**
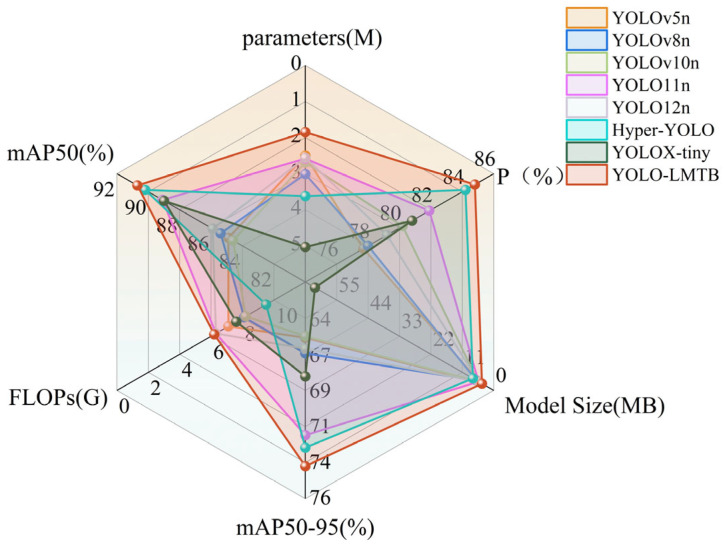
Comparison of Tea Bud Detection Performance Between Mainstream Models and Improved Models.

Figure 6 presents a comparison of the detection performance among YOLOv11n, Hyper-YOLO, and the proposed YOLO-LMTB model. As illustrated, YOLOv11n and Hyper-YOLO exhibit varying degrees of missed detections and false positives when handling occluded and densely distributed targets. In particular, both models detect significantly fewer extremely small tea buds compared to YOLO-LMTB. Moreover, their confidence scores are mostly lower across all scenarios than those of YOLO-LMTB. These results indicate that YOLO-LMTB achieves superior detection performance in challenging environments characterized by dense distributions, partial occlusions, and significant scale variations among tea buds. Figure 7 provides an intuitive visualization via a radar chart based on the experimental data from Table 1, further emphasizing the outstanding performance of YOLO-LMTB across multiple metrics.

Among all the evaluated methods, the proposed YOLO-LMTB demonstrates the strongest overall performance. Compared to the baseline YOLOv11n model, it achieves improvements of 2.9% in precision, 1.6% in mAP50, and 2.0% in mAP50-95. With the exception of mAP50-95, which is marginally lower than that of YOLOv11m, all other metrics attain the highest values among the compared models. Furthermore, YOLO-LMTB enhances detection accuracy while simultaneously achieving model lightweighting: the parameter count and model size are reduced by 28.3% and 22.6%, respectively, with FLOPs remaining nearly unchanged—indicating no additional computational overhead. A comparison with YOLOv11s and YOLOv11m shows that YOLO-LMTB outperforms YOLOv11s in accuracy while using only approximately 20% of its parameters and model size. Although the mAP50-95 of YOLO-LMTB is 1.3% lower than that of YOLOv11m, it excels in all other performance metrics. These results confirm that the proposed method delivers detection performance comparable to YOLOv11m, but at substantially reduced computational cost.

To further validate the effectiveness of the proposed improvement strategy, the aforementioned enhancements were integrated into YOLOv8n and YOLOv10n—structures similar to YOLOv11—and designated as YOLOv8n-LMTB and YOLOv10n-LMTB, respectively. Relevant comparative experimental data are detailed in Table 3.

As shown in the experimental data from Table 3, our improved strategy is equally applicable to YOLOv8n and YOLOv10n, achieving significant performance gains that validate the effectiveness and broad applicability of the enhancement approach. Although both YOLOv8 and YOLOv10 models employing the improved scheme demonstrate considerable improvements, overall, YOLO-LMTB retains superior performance metrics. Therefore, we retain YOLO-LMTB as the final model.

### 3.4. Ablation Experiment

#### 3.4.1. Application on the Self-Built Tea Bud Dataset

To validate the effectiveness of the proposed improvements, we conducted an ablation study on our self-built tea bud dataset to evaluate the contributions of the MERCA, DHTST, and BiFPN modules. Each component was added incrementally to assess its individual impact. All experiments were performed under identical hardware, software, and parameter settings to ensure scientific rigor and comparability. The results are summarized in Table 4.

When any combination of these modules is used, the strengths of each module are effectively exploited, further improving the model’s detection performance. Ultimately, the integration of all three modules—MERCA, DHTST, and BiFPN—combines the advantages of high detection accuracy and a lightweight architecture, resulting in the best overall performance. The proposed YOLO-LMTB model achieves a precision of 84.8%, with mAP50 and mAP50-95 reaching 90.7% and 73.9%, respectively. Furthermore, the total parameter count and model size are reduced to 1.85 million and 4.1 MB.

**Figure 8:**
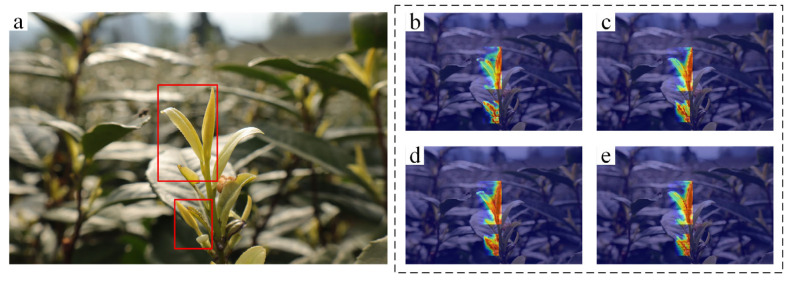
Comparison of Model Improvement Heatmap Results. (**a**) Ground truth, (**b**) YOLO11n model, (**c**) With MERCA, (**d**) With MERCA and DHTST, (**e**) With MERCA, DHTST, and BiFPN. The red box in area (**a**) indicates the actual target region.

To more intuitively demonstrate the effectiveness of the proposed modules, we employed Grad-CAM [33] to visualize and compare feature maps across different model configurations, as shown in Figure 8. In the heatmaps, color intensity corresponds to the model’s attention level, with redder regions indicating higher focus.

The results show that as each proposed module is incrementally integrated, the model exhibits increasingly concentrated attention on the core features of tea bud targets. The attention areas become more precise and less dispersed, effectively suppressing interference from background elements. The visualizations confirm that the enhanced model not only strengthens its focus on discriminative regions of tea buds but also mitigates distractions from complex backgrounds, thereby significantly improving feature extraction capability.

#### 3.4.2. Application on Public Tea Bud Datasets

To further validate the generalization capability of the improved solution, we conducted ablation experiments on the publicly available tea bud dataset in addition to our self-built dataset. The ablation study results for this dataset are presented in Table 5.

As shown in Table 5, our proposed improvement modules remain effective on public datasets, significantly enhancing the model’s detection performance. They not only improve detection accuracy but also reduce computational costs. Compared to the baseline model, the improved approach achieves 75.9% precision on public datasets, with mAP50 and mAP50-95 increasing by 3.5% and 3.1%, respectively. The number of model parameters was reduced by 28.2%, and the model size was decreased by 21.2%. These experimental results further validate the effectiveness and generalization capability of the improved approach.

## 4. Conclusions

To enable precise and efficient detection of tender tea buds and promote intelligent tea harvesting, this paper thoroughly considers the growth characteristics of tea buds and the challenges of the detection task, proposing a lightweight object detection model named YOLO-LMTB based on YOLOv11n. First, the novel MERCA module fully leverages cross-scale features while emphasizing edge information of tea buds, effectively enhancing the model’s ability to handle multi-scale targets in real detection scenarios. Second, the designed DHTST module improves feature representation through dynamic activation and token statistical recalibration, enabling more accurate target localization in complex environments. Finally, the introduced BiFPN module optimizes multi-scale feature fusion, enhances global information aggregation, and reduces computational overhead. Results on both our self-built tea bud dataset and public datasets demonstrate that YOLO-LMTB outperforms various YOLO variants in core metrics including precision, mAP50, and parameter efficiency. Furthermore, our proposed improvements exhibit strong generalization capabilities and effectiveness. This model provides a scientifically reliable technical solution for tea bud detection in smart agriculture and contributes to the intelligent advancement of tea cultivation.

Although the proposed model achieves accurate tea bud detection while maintaining a lightweight architecture, several limitations remain: (1) Current tea bud datasets are primarily self-collected, and there is a lack of high-quality public datasets covering diverse tea varieties. This scarcity considerably impedes progress in intelligent tea processing. In future work, we plan to construct a more comprehensive dataset including multiple tea varieties from various regions to improve generalization and diversity; (2) While the model exhibits improved performance in detecting extremely small objects, there is still considerable room for enhancement. Further optimizations will be pursued to strengthen detection robustness in highly complex scenarios; (3) Although theoretical evaluations confirm the model’s lightweight characteristics, practical deployment on edge devices has not yet been conducted. Future work will include real-world deployment and optimization of inference speed, energy efficiency, and other operational metrics on embedded platforms.

## Figures and Tables

**Table 1:** Experimental results of different replacement schemes for MERCA modules.

Model	P (%)	mAP50 (%)	mAP50 -95 (%)	Parameters (M)	FLOPs (G)	Model Size (MB)
YOLO11n	81.9	89.1	71.9	2.58	6.3	5.3
YOLO11n-MERCA-Backbone	80.4	90.0	73.0	2.53	6.3	5.3
YOLO11n-MERCA-Neck	78.9	88.6	69.6	2.57	6.4	5.4
YOLO11-MERCA-All	81.5	89.1	70.3	2.52	6.3	5.5

**Table 2:** Experimental Results of Mainstream Models on self-built dataset.

Model	P (%) *	mAP50 * (%)	mAP50 * -95 (%)	Parameters (M)	FLOPs (G)	Model Size (MB)
YOLOv5n	77.7 (0.13)	84.9 (0.16)	65.6 (0.17)	2.50	7.1	5.0
YOLOv8n	78.0 (0.22)	85.4 (0.13)	66.6 (0.14)	3.01	8.1	6.0
YOLOv10n	80.2 (0.17)	84.6 (0.21)	65.5 (0.25)	2.69	8.2	5.5
YOLO11n	81.9 (0.25)	89.1 (0.14)	71.9 (0.13)	2.58	6.3	5.3
YOLO11s	82.4 (0.13)	90.1 (0.33)	73.7 (0.21)	9.41	21.3	19.2
YOLO11m	84.4 (0.13)	89.9 (0.17)	75.2 (0.13)	20.03	67.6	40.6
YOLO12n	79.2 (0.1)	85.9 (0.08)	66.3 (0.17)	2.56	6.3	5.3
Hyper-YOLO	84.2 (0.16)	90.2 (0.17)	72.7 (0.22)	3.62	9.5	7.3
YOLOX-tiny	80.8 (0.19)	89.0 (0.22)	68.1 (0.21)	5.03	7.6	62.6
YOLO-LMTB	84.8 (0.13)	90.7 (0.22)	73.9 (0.19)	1.85	6.2	4.1

* All data are the results of averaging three experiments, with the standard deviations in parentheses.

**Table 3:** Comparison of the improvement strategies in YOLOv8n, YOLOv10n and YOLO11n.

Model	P (%)	mAP50 (%)	mAP50 -95 (%)	Parameters (M)	FLOPs (G)	Model Size (MB)
YOLOv8n	78.0	85.4	66.6	3.01	8.1	6.0
YOLOv8n-LMTB	82.7	89.6	72.1	1.84	6.6	3.9
YOLOv10n	80.2	84.6	65.5	2.69	8.2	5.5
YOLOv10n-LMTB	81.6	88.8	70.6	1.51	5.0	3.9
YOLO11n	81.9	89.1	71.9	2.58	6.3	5.3
YOLO-LMTB	84.8	90.7	73.9	1.85	6.2	4.1

**Table 4:** Ablation research results of each module of YOLO-LMTB on the self-built dataset.

Baseline	MERCA	DHTST	BiFPN	P (%)	mAP50 (%)	mAP50 -95 (%)	Parameters (M)	FLOPs (G)	Model Size (MB)
✓	**×**	**×**	**×**	81.9	89.1	71.9	2.58	6.3	5.3
✓	✓	**×**	**×**	80.4	90.0	73.0	2.53	6.3	5.3
✓	**×**	✓	**×**	83.1	90.1	72.2	2.56	6.3	5.2
✓	**×**	**×**	✓	83.4	90.1	72.9	1.92	6.3	4.0
✓	✓	✓	**×**	83.8	90.8	73.2	2.51	6.3	5.3
✓	✓	**×**	✓	84.4	90.8	73.5	1.87	6.3	4.1
✓	**×**	✓	✓	84.4	90.8	73.4	1.91	6.3	4.0
✓	✓	✓	✓	84.8	90.7	73.9	1.85	6.2	4.1

✓ indicates that the module is selected, **×** indicates that the module is not selected.

**Table 5:** Ablation research results of each module of YOLO-LMTB on public datasets.

Baseline	MERCA	DHTST	BiFPN	P (%)	mAP50 (%)	mAP50 -95 (%)	Parameters (M)	FLOPs (G)	Model Size (MB)
✓	**×**	**×**	**×**	73.5	72.8	52.0	2.58	6.3	5.2
✓	✓	**×**	**×**	73.1	74.8	54.2	2.53	6.3	5.3
✓	**×**	✓	**×**	74.1	74.6	54.1	2.57	6.3	5.2
✓	**×**	**×**	✓	73.9	74.7	53.9	1.92	6.3	4.0
✓	✓	✓	**×**	75.2	75.6	54.6	2.51	6.3	5.3
✓	✓	**×**	✓	75.3	76.1	54.8	1.87	6.3	4.1
✓	**×**	✓	✓	75.7	76.0	54.9	1.91	6.3	4.0
✓	✓	✓	✓	75.9	76.3	55.1	1.85	6.2	4.1

✓ indicates that the module is selected, **×** indicates that the module is not selected.

## Data Availability

All data included in this study are available upon request by contact with the corresponding author.
